# The blood glucose trajectories among non-diabetic patients with total joint arthroplasty: clinical characteristics and predictors

**DOI:** 10.3389/fmed.2026.1820392

**Published:** 2026-06-25

**Authors:** Peifang Li, Guihua Miao, Qin Wang, Liqun Wang, Ning Ning, Jiali Chen

**Affiliations:** Department of Orthopedic Surgery, West China Hospital/West China School of Nursing, Sichuan University, Chengdu, China

**Keywords:** blood glucose trajectories, GBTM, LASSO, random forest model, TJA patients

## Abstract

**Objective:**

To classify the blood glucose trajectories using group-based trajectory models (GBTM) and construct a random forest model to identify predictive factors to forecast different blood glucose trajectories in non-diabetic patients with total joint arthroplasty (TJA).

**Methods:**

A prospective observational study was carried out from September 2022 to May 2024 in West China Hospital, Sichuan University. Patients without diabetes mellitus aged 18 to 80 years who underwent unilateral elective primary TJA for end-stage osteoarthritis were included in this clinical study. We measured the blood glucose level of TJA patients on preoperative 1 day and postoperative days (PODs) 0 to 2 to identify the subgroups of blood glucose trajectories in TJA patients by GBTM. Meanwhile, we collected the socio-demographic characteristics, disease-related data, results from routine blood tests, and information regarding perioperative drug use to analyze the predictors of different subgroups of blood glucose trajectories in patients with TJA.

**Results:**

Three distinct groups emerged: Group 1—Normal blood glucose, stable (49.5%); Group 2—Postoperative blood glucose slightly increased with minor fluctuations (41.7%); and Group 3—Hyperglycemia with significant fluctuations (8.8%). Those predictors integrated by random forest (RF) model were age, red blood cell (RBC) one day post-surgery, hypertension, diclofenac, intraoperative blood transfusion volume, and Huaxi Emotional-distress Index (HEI). The RF model achieved an overall accuracy rate of 78.3% (95% CI: 73.1–83.0%), with a Kappa coefficient of 0.600.

**Conclusion:**

In this study, we found three blood glucose trajectories subgroups of TJA patients in the perioperative period using GBTM and analyzed their predictors by a RF model. However, at the current classification threshold, the model has limited ability to identify patients with hyperglycaemic trajectories.

## Background

Over 100,000 joint replacements are performed in Australia each year. The rate of joint replacement continues to increase in Australia by 73.7% for hip replacements, 111.5% for knee replace ment since 2003.

With demographic changes and improvements in living standards, healthcare, nutrition and education, the number of elderly patients undergoing total joint arthroplasty (TJA) is steadily increasing (1). The incidence of joint replacement procedures is over 1 million each year in the United States. By 2030, the incidence of primary total hip and knee arthroplasties is projected to rise by 71 and 85%, respectively (2). TJA is a highly effective intervention for the treatment of end-stage arthritis (3). This procedure can effectively relieve joint pain, restore joint function and stability, correct deformities, and improve the quality of life in patients.

However, a successful surgery does not necessarily equate to treatment success. With advances in prosthetic materials and surgical techniques, advanced age is no longer a contraindication for TJA (4, 5). Elderly patients frequently present with comorbidities such as hypertension, diabetes mellitus, and cardiovascular diseases, which increase the risk of postoperative complications following TJA. A recent study reported that the incidence of complications after TJA was 30.42% (4). These complications primarily include infection, venous thromboembolism (VTE), delirium, dislocation, and periprosthetic fractures (1, 4). As a major surgery, TJA exposes patients to metabolic stress, resulting in blood glucose fluctuations, a critical risk factor for postoperative complications in orthopedic surgery (6, 7).

Postoperative hyperglycemia in patients undergoing TJA results from perioperative pharmacotherapy, impaired glucose tolerance, glucocorticoid administration, and physiological stress responses post-surgery. The incidence of hyperglycemia in TJA patients after surgery is 40% (8), leading to serious complications, including infection, venous thromboembolism, poor functional outcomes, extended hospitalization, escalated healthcare expenses, and elevated readmission risks (3).

The utilization of blood glucose variability as a proxy for glycemic control has gained prominence in recent years (7, 9). Rapid fluctuations in blood glucose levels can elevate oxidative stress more significantly than sustained hyperglycemia (10). Sharp and short-term fluctuations in blood glucose levels can affect immune function, potentially impairing the host’s ability to resist infection (10). Therefore, elevated blood glucose variability during the perioperative period is associated with surgical site and periprosthetic joint infections, elevated complication rates, and mortality (7, 9).

Most existing researches on perioperative hyperglycemia or blood glucose variation primarily focuse on blood glucose values while neglecting the temporal aspect of blood glucose changes. Additionally, the analysis of blood glucose variations across all study participants obscures the heterogeneity of blood glucose levels among different patients undergoing TJA. Group-based trajectory models (GBTM) may provide a promising alternative to summarize the trends in perioperative blood glucose changes for TJA patients and can identify individuals with similar longitudinal response patterns.

Therefore, this study aims to (1) classify the blood glucose trajectories of TJA patients during the perioperative period using GBTM and analyze the characteristic differences in these trajectories among various patient groups, and (2) construct a random forest (RF) model to identify predictive factors to forecast different blood glucose trajectories in TJA patients. By utilizing these predictors, medical staff can anticipate the blood glucose trajectories of patients, which is crucial for providing precise dietary guidance, managing blood glucose levels, preventing complications, and facilitating early rehabilitation during the perioperative period in patients with TJA.

## Materials and methods

### Design

A prospective observational study was carried out from September 2022 to May 2024, with the methods and results reported following the GRoLTS-Checklist, a guideline for latent class trajectory studies in West China Hospital, Sichuan University (11). This clinical study has been approved by the Ethics Committee on Biomedical Research of our university (20211170). Informed consent was obtained from all participants.

### Patients

Patients aged 18 to 80 years who underwent unilateral elective primary TJA for end-stage osteoarthritis were included in this clinical study. Those who had diabetes mellitus regardless of glycemic control, severe liver or kidney failure, or inflammatory diseases, were excluded from participation.

### Perioperative management

A standardized clinical protocol was implemented for each patient undergoing TJA (12). Patients were instructed in preoperative breathing techniques, rehabilitation exercises, positioning maneuvers, and the use of mobility aids. Intravenous tranexamic acid was administered at a dose of 20 mg/kg 10 min before incision, with additional 1 g doses given intravenously at 3, 6, and 24 h postoperatively. A multimodal analgesic protocol was initiated upon admission, with all patients receiving imrecoxib 100 mg twice daily. Prior to wound closure, patients received standardized local infiltration analgesia comprising 200 mg ropivacaine (0.25%) in deep and superficial tissues, including the superficial fascia and subcutaneous tissues. Following surgery, patients undergoing THA continued imrecoxib 100 mg twice daily, while those undergoing TKA received imrecoxib 100 mg twice daily and oxycodone 10 mg every 12 h. An intramuscular injection of morphine (10 mg) was administered if the numerical rating scale (NRS) pain score exceeded 5.

Patients were administered a standardized thromboembolic prophylaxis regimen, including immediate use of intermittent pressure devices (IPC) upon returning to the ward post-surgery, a subcutaneous injection of 2000 IU enoxaparin at 8 h postoperatively, followed by a daily dose of 4,000 IU. Additionally, patients were prescribed 10 mg of rivaroxaban for 10 days post-discharge. Intraoperatively, 10 mg of dexamethasone was intravenously administered to prevent postoperative vomiting. Metoclopramide was given intramuscularly to patients experiencing postoperative nausea and vomiting (PONV). Ankle pump exercises were performed in bed on the day of the operation, while on the first day, patients were assisted in mobilizing for functional exercises to prevent complications.

### Data collection

Considering the clinical relevance and risk factors associated with blood glucose fluctuations documented in the literature, the researchers designed a self-administered questionnaire. This instrument encompassed socio-demographic characteristics, disease-related data, results from routine blood tests, and information regarding perioperative drug use. (1) Socio-demographic data included age, gender, weight, height, nationality, education level, marital status, and occupation. (2) Clinical data comprised diagnosis, disease duration, pain at admission, self-care ability score, mood, comorbidities (hypertension, heart disease, cerebrovascular disease), operation duration (in minutes), intraoperative blood transfusion volume, and intraoperative blood loss. (3) Complete blood count parameters 1 day post-surgery included red blood cell (RBC) count, hemoglobin level, leukocyte count, platelet count, neutrophil count, and lymphocyte count. During the perioperative period, the responsible nurse collected the patient’s blood samples as per the doctor’s orders and sent them to the hospital’s laboratory. The laboratory issued the relevant blood test result reports, and the researcher extracted the required data from the blood test reports. (4) Information on drug use during the perioperative period addressed whether opioid medications, diclofenac, alprazolam, iron supplements, or anti-anxiety drugs were administered postoperatively.

Sociodemographic data were gathered within 6 hours of the patient’s admission. As treatment progressed, information regarding the patient’s disease, intraoperative conditions, test results, and perioperative medications was collected. Pain levels and self-care ability were assessed using the NRS and the Activities of Daily Living (ADL) scale, respectively. The emotional disorder at admission was screened by the Huaxi Emotional-distress Index (HEI) (13), a 9-item questionnaire to detect patients’ emotional disturbances especially in the busy non-psychiatric clinical settings in China. A complete blood count was obtained on the first day of admission and again on the morning following the operation.

### Blood glucose

We measured the blood glucose levels of TJA patients on the day before operation and postoperative days 0 to 2. On the day before operation, blood glucose was measured four times, including fasting blood glucose prior to breakfast and postprandial blood glucose 2 hours after each of the three meals (T1-4). On the day of operation, blood glucose was assessed three times: before breakfast (T5), immediately upon returning to the ward (T6), and before bedtime (T7). On the postoperative day 1 (T8-T11) and 2 (T12-T15), blood glucose was measured four times as the day before operation. All blood glucose levels were determined using fingertip rapid blood glucose testing (CONTOUR®PLUS ONE, Bayer).

### Statistical analyses

Continuous variables following a normal distribution were presented as mean (SD), while those deviating from normality were depicted as medians with interquartile ranges (IQRs). The normality of data was assessed using the Shapiro–Wilk test, and homogeneity of variance was evaluated using the Bartlett test. Categorical variables were expressed as absolute values and percentages. The patients’ clinical characteristics were statistically summarized using SPSS 27.0 software.

Longitudinal data is susceptible to issues related to missing data. In this study, the random forest method was employed to interpolate the missing data, utilizing the missForest package in R version 4.3.0.

GBTM is an interpretive modeling method that clusters longitudinal data using a finite mixture model to uncover distinct trajectory categories. The strength of GBTMs lies in their ability to identify population heterogeneity or specific subgroups exhibiting similar patterns over extended periods. Thus, the categorization of patient groups is driven by data rather than clinical considerations (14). In this study, GBTM was employed to identify potential individual subpopulations with comparable trends in blood glucose level changes during the perioperative period. We tested models comprising two to six groups, incorporating constant, linear, quadratic, or cubic terms. Model selection was guided by the Bayesian Information Criterion (BIC), with the model yielding the lowest BIC value deemed optimal for the number and form of trajectory categories. Trajectory attribution was determined based on individual posterior probabilities, and the average posterior probability along with the correct classification odds ratio were calculated to assess the model’s intrinsic validity. GBTM was implemented using R version 4.4.1, specifically through the gbmt package in the statistical software.

Because candidate variables exist in high-dimensional complex collinearity, we used the Lasso regression model to select the most useful prognostic risk factors of the blood glucose trajectories of TJA patients. To evaluate the stability of the LASSO-selected predictors, a bootstrap resampling procedure was conducted with 1,00 iterations. At each iteration, a bootstrap sample was drawn with replacement, and the LASSO model was refitted using the lambda value determined by the 1SE criterion from the full-sample cross-validation. The selection frequency—defined as the proportion of bootstrap iterations in which a given variable retained a non-zero coefficient—was computed for each candidate predictor. This approach is particularly valuable in clinical prediction models where interpretability is crucial.

The variables selected through Lasso regression were utilized to develop a RF model. Internal validation was performed by resampling 500 times using the Bootstrap method. The model was constructed, and its performance was assessed using the entire dataset. The random forest prediction model ranks the predictors from highest to lowest based on the average decrease in the Gini coefficient. Subsequently, prediction accuracy, sensitivity, specificity, and the area under the receiver operating characteristic (ROC) curve were calculated to evaluate model performance. Statistical analyses for this section were conducted using R (version 4.1.3) with the “randomForest” software package.

We deliberately chose a two-step approach of Lasso regression followed by RF classification to select variables for several methodological reasons. First, Lasso regression provides a statistically rigorous framework for variable selection with clear statistical criteria (cross-validation for *λ* selection), whereas random forest variable importance measures can be biased toward variables with more categories or larger measurement scales. Second, the Lasso step reduces dimensionality and eliminates collinearity, creating a more stable and interpretable predictor set for the subsequent random forest model. Finally, by limiting the random forest to stable, clinically meaningful predictors identified by Lasso, we improved model interpretability—a critical consideration for clinical decision support tools.

## Results

### Demographic and clinical characteristics

Initially, 276 patients who met the inclusion and exclusion criteria were investigated. Of these, 36 patients were excluded because their blood glucose levels were not monitored either before or after the operation due to their absence for examinations outside. Ultimately, 240 patients were included in this study. Among these participants, 69.2% were women, and the median age was 62.5 years. Additionally, 47.1% of the patients were diagnosed with avascular necrosis of the femoral head or femoral neck fracture. Furthermore, 79.2% of the patients reported mild pain, as indicated by a NRS score of 1–3. Other available demographic data are presented in Table 1.

**Table 1:** Sociodemographic and clinical characteristics of the TJA patients.

Variable	*N* = 240
Age (year, $\bar{x}$ ±*s*)	62.5 ± 13.0
Gender (*n*, %)	
Male	74 (30.8)
Female	166 (69.2)
Weight (kg, $\bar{x}$ ±*s*)	62.9 ± 10.8
Height (cm, $\bar{x}$ ±*s*)	158.9 ± 7.9
Nation (*n*, %)	
Han	223 (92.9)
Others	17 (7.1)
Marriage (*n*, %)	
Married	193 (80.4)
Spinsterhood	11 (4.6)
Divorced	7 (2.9)
Widowed	29 (12.1)
Education (*n*, %)	
Illiteracy	96 (40.0)
Primary school	66 (27.5)
Middle school	39 (16.3)
College or above	39 (16.3)
Occupation (*n*, %)	
No	64 (26.7)
Manual workers	114 (47.5)
Mental worker	62 (25.8)
Pain at admission (NRS) (*n*, %)	
0	49 (20.4)
1	41 (17.1)
2	133 (55.4)
3	16 (6.7)
4	1 (0.4)
Self-care ability score (ADL) (score, $\bar{x}$ ±*s)*	85.1 ± 16.9
Huaxi emotional-distress index (HEI) (*n*, %)	
No	237 (98.7)
Yes	3 (1.3)
Disease duration [Year, *M*(*Q*_1_, Q_3_)]	8 (3, 12)
Diagnosis (*n*, %)	
Knee Osteoarthritis	64 (26.7)
Knee rheumatoid arthritis	33 (13.8)
Developmental dysplasia of the hip	30 (12.5)
Necrosis of the femoral head/femoral neck fracture	113 (47.1)
Hypertension (*n*, %)	
Yes	86 (35.8)
No	154 (64.2)
Heart disease (*n*, %)	
Yes	6 (2.5)
No	234 (97.5)
Cerebrovascular disease (*n*, %)	
Yes	6 (2.5)
No	234 (97.5)
Operation duration (min, $\bar{x}$ ±*s*)	94.3 ± 56.2
Intraoperative blood transfusion volume (mL) (*n*, %)	
0	233 (97.1)
50	2 (0.8)
200	1 (0.4)
250	3 (1.3)
400	1 (0.4)
Intraoperative blood loss (mL, $\bar{x}$ ±*s*)	133.0 ± 110.3
Opioid medications (*n*, %)	
Yes	195 (81.2)
No	45 (18.8)
Difene (*n*, %)	
Yes	102 (42.5)
No	138 (57.5)
Alprazolam (*n*, %)	
Yes	151 (62.9)
No	89 (37.1)
Iron supplements (*n*, %)	
Yes	20 (8.3)
No	220 (91.7)
Anti-anxiety drugs (*n*, %)	
Yes	18 (7.5)
No	222 (92.5)
RBC (×10^12^/L, $\bar{x}$ ±*s*)	3.9 ± 0.6
Hb (g/L, $\bar{x}$ ±*s*)	118.9 ± 14.8
WBC (×10^9^/L, $\bar{x}$ ±*s*)	11.1 ± 9.3
PLT (/L, $\bar{x}$ ±*s*)	179.5 ± 55.2
ANC [×10^9^/L, *M*(*Q*_1_, Q_3_)]	8.5 (6.9, 10.4)
ALC [×10^9^/L, *M*(*Q*_1_, Q_3_)]	1.0 (0.8, 1.3)

### Blood glucose trajectory subgroups by GBTM

**Figure 1:**
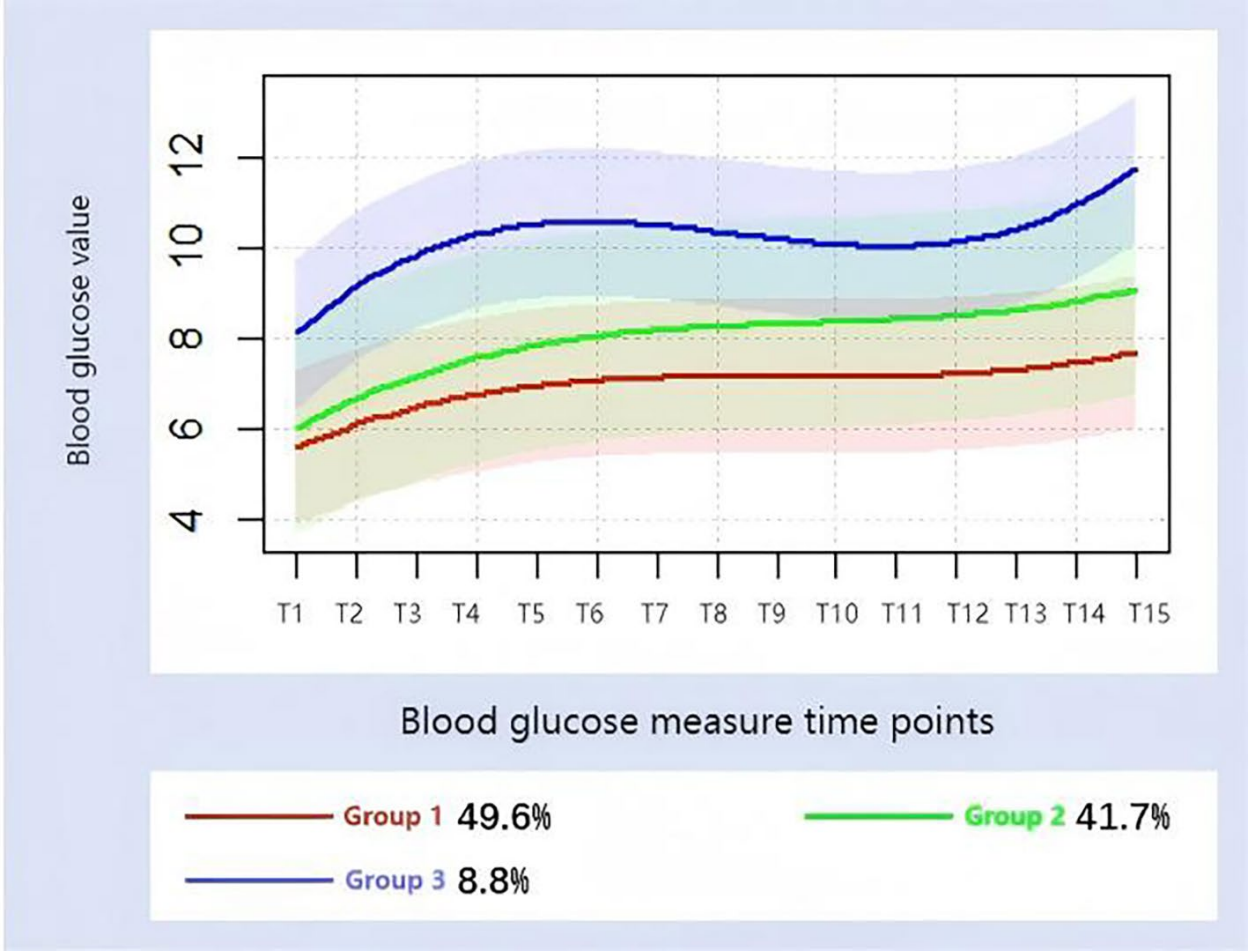
The blood glucose trajectory groups identified by group-based trajectory modeling. The percentage represented the proportion of patients in each trajectory group.

To determine the optimal number of latent trajectory groups, this study successively fitted latent class growth mixture models with the number of groups ranging from 1 to 5. The best model was selected by comprehensively considering information criteria, model degeneracy, and the clinical interpretability of the trajectories. The comparison of information criteria for each candidate model is presented in Supplementary Table 1. As shown in Supplementary Table 1, when the number of groups increased from 1 to 3, all information criterion values decreased significantly, indicating a continuous improvement in model fit. When the number of groups increased to 4, the Akaike Information Criterion (AIC), Consistent Akaike Information Criterion (CAIC), and Hannan-Quinn Information Criterion (HQIC) further decreased slightly and reached their minimum values. However, the BIC and CAIC values showed a slight increase for the 4-group model compared with the 3-group model. When the number of groups increased to 5, most information criterion values either rebounded or plateaued relative to those of the 4-group model. Overall, both the 3-group and 4-group models achieved a favorable balance between goodness-of-fit and model complexity, making them candidate models. To further evaluate the 3-group and 4-group models, we visually compared their trajectory patterns (Figure 1 and (Supplementary Figure 1). The results showed that in the 4-group model, two trajectories (Group 3 and Group 4) exhibited highly similar trends over time, suggesting potential over-specification of the number of groups, i.e., these two subgroups could reasonably be combined into a single trajectory. In contrast, each trajectory in the 3-group model demonstrated clearer distinction and greater clinical interpretability. Based on the marginal improvement in information criteria, the distinctness of the trajectories, and the principle of model parsimony, the model with three subgroups was ultimately selected as the optimal latent trajectory solution in this study.

Figure 1 illustrates the perioperative blood glucose trajectory groups identified through GBTM. Three distinct groups emerged: Group 1—Normal blood glucose, stable (49.5%); Group 2—Postoperative blood glucose slightly increased with minor fluctuations (41.7%); and Group 3—Hyperglycemia with significant fluctuations (8.8%). The blood glucose measurements in the normal group remained largely within the normal range. The group exhibiting slightly elevated blood glucose levels demonstrated essentially normal values the day prior to surgery, with a modest increase observed postoperatively, characterized by relatively minor fluctuations. Conversely, the hyperglycemic group maintained levels above the normal range both before and after surgery, accompanied by considerable fluctuations.

### Factor selection

**Figure 2:**
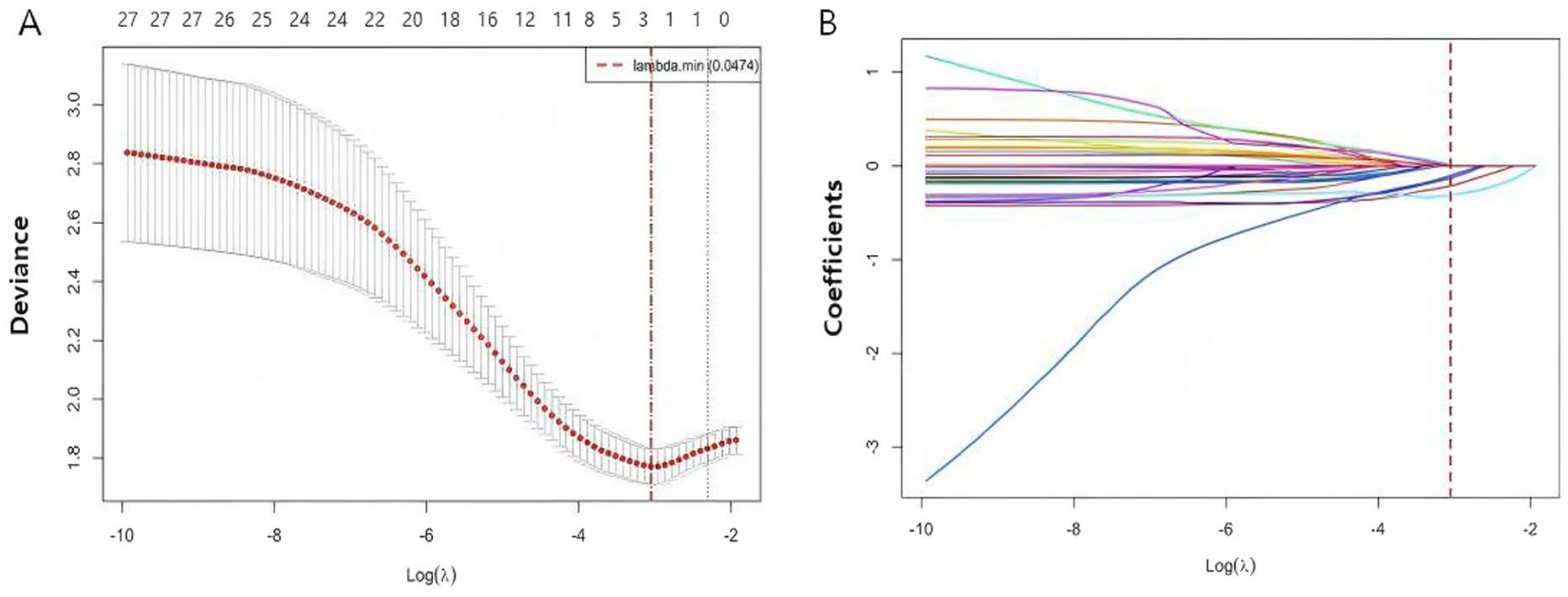
LASSO regression analysis to select prognostic factors for the blood glucose trajectory subgroups. **(A)** The cross-validation results. The optimal lambda was selected by tenfold cross-validation. Two dashed line were displayed at the optimal values in the minimum criterion and the 1SE of the minimum criterion. At our results, the optimal lambda was in 1SE of the minimum criterion. Six variables were selected when log (*λ*) = 0.047. **(B)** LASSO coefficient profiles of the 6 variables. The figure B showed their coefficients of each variable when the corresponding number of variables with different the values of Log lambda were included in the LASSO logistic regression model. The dashed line showed the coefficients of 6 variables selected by tenfold cross-validation.

The bootstrap stability analysis revealed that the six retained predictors demonstrated variable selection frequencies ranging from 0.69 to 0.99 across 100 bootstrap iterations. Specifically, HTN (0.99), age (0.98), Blood Transfusion (0.96), Difen (0.95) and RBC (0.90) exhibited high selection frequencies (>0.80), indicating robust selection stability. Huaxi moods index (0.69) showed moderate to high selection frequencies. Lasso regression analysis was employed to identify predictive factors for the blood glucose trajectory groups (Figure 2). The tuning parameter (*λ*) was determined using tenfold cross-validation (Figure 2A). Two dotted vertical lines were drawn at the optimal lambda values, based on the minimum criteria and the 1 standard error of the minimum criteria (the 1SE criteria). Specifically, log(λ) = 0.047 was selected according to the 1SE criteria. A coefficient profile plot was generated (Figure 2B), with a dotted vertical line indicating the 1SE criteria, where the optimal lambda yielded six nonzero coefficients. Consequently, the six predictive factors associated with the blood glucose trajectory groups were identified as age, hypertension, diclofenac, RBC one day post-surgery, intraoperative blood transfusion volume, and HEI. The corresponding non-zero coefficients for these factors are −0.2135, −0.3098, −0.1971, −0.1312, −0.1043, and 0.0113, respectively.

### RF model

**Figure 3:**
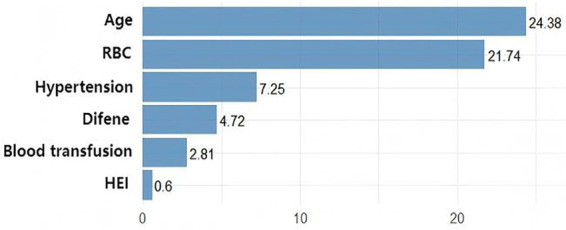
Ranking of variable importance by the random forest model.

The RF model was developed using the six variables identified through Lasso regression analysis. The ranking of variable importance was determined based on the average decrease in the Gini coefficient for each influencing factor within the model, resulting in the following order: age, RBC one day post-surgery, hypertension, diclofenac, intraoperative blood transfusion volume, and HEI (Figure 3). These variables are considered potential key predictors for distinguishing the blood glucose trajectory subgroups.

### RF models’ performance

The RF model demonstrated strong overall discriminative performance. The model achieved an overall accuracy rate of 78.3% (95% CI: 73.1–83.0%), with a Kappa coefficient of 0.600, indicating moderate agreement between the model’s predictions and the actual classifications. Additionally, the weighted average F1 score of 0.771 reflects the model’s robustness in addressing class imbalance issues.

**Figure 4:**
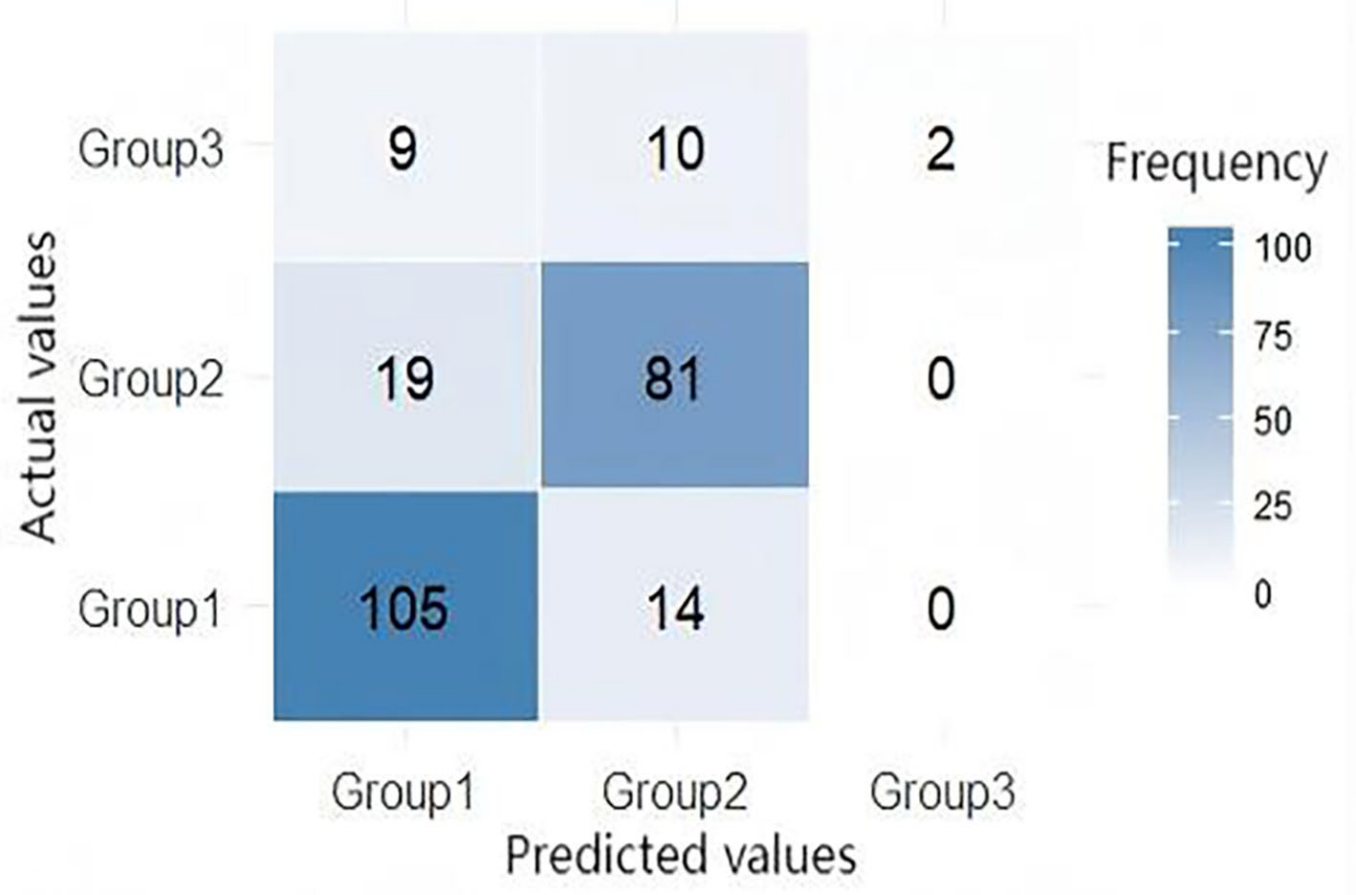
Confusion matrix of the random forest model in the patient’s blood glucose trajectories.

**Figure 5:**
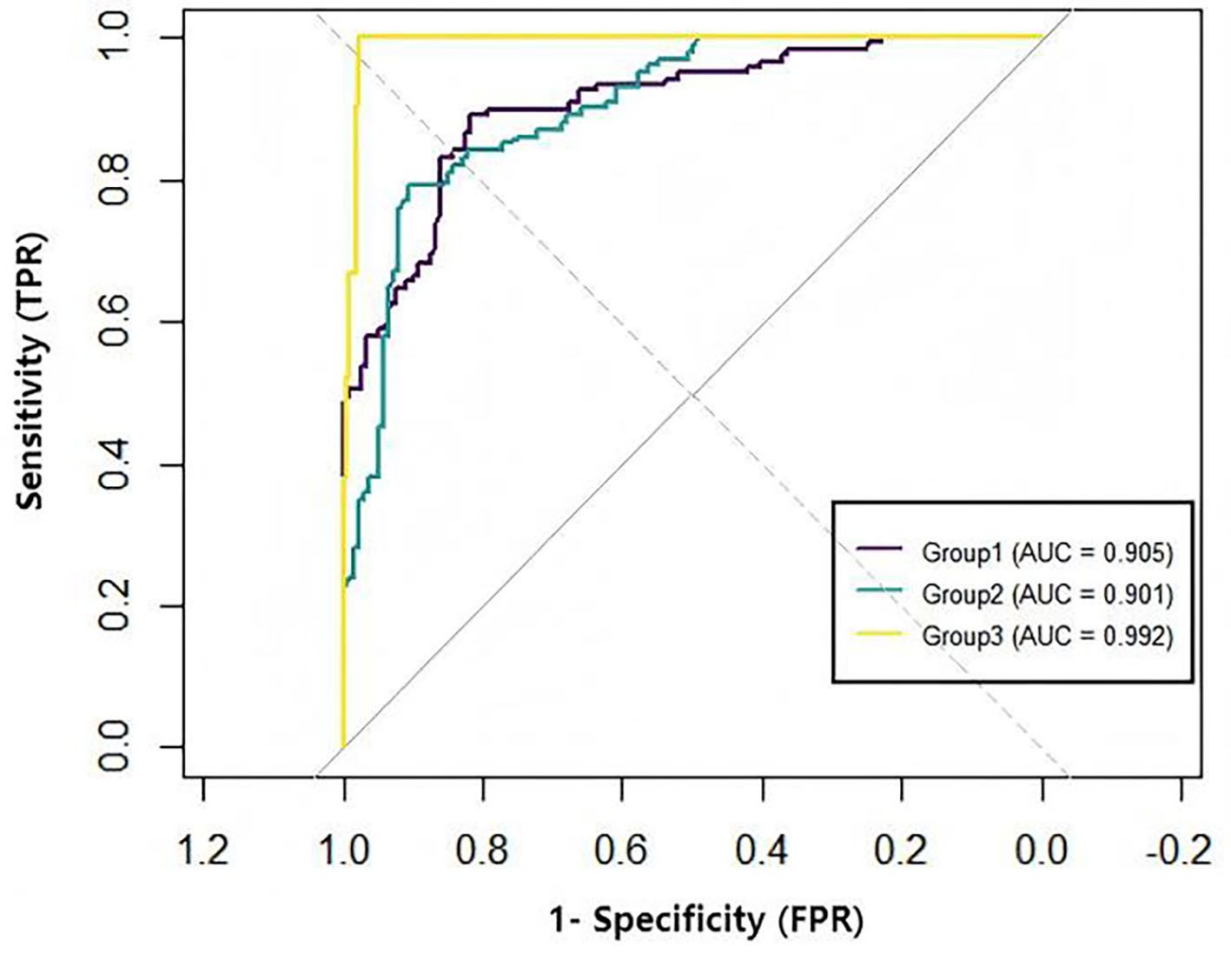
The ROC curves of the random forest model in the patient’s blood glucose trajectories.

The specific discriminative performance of each trajectory group is illustrated in the comprehensive confusion matrix heatmap (Figure 4) and the ROC curve (Figure 5). Detailed performance indicators for the RF model are presented in Table 2. The model exhibited the highest recognition capability for Group 1 (normal blood glucose group), achieving an area under the curve (AUC) of 0.905, with sensitivity and specificity values of 0.882 and 0.769, respectively. For Group 2 (the group with slightly elevated blood glucose), the model also demonstrated stable performance, attaining an AUC of 0.901, with relatively balanced precision and recall rates. In contrast, the model’s recognition of Group 3 (hyperglycemia group) revealed high specificity (1.000) but low sensitivity (0.095). Although the AUC value reached 0.992, indicating excellent sorting ability, the extremely low recall rate suggests a significant missed judgment rate for this group. This phenomenon may be attributed to category imbalance stemming from the relatively small sample size (*n* = 21) in the hyperglycemia group. In addition, we attempted to re-evaluate the current model using LOOCV. However, the results showed that the overall performance metrics of the model (e.g., accuracy and Kappa coefficient) did not reach the desired level (Supplementary Table 2). We suspect that this may be due to the relatively limited sample size, severe class imbalance, and high inter-individual heterogeneity in our dataset. Under these circumstances, LOOCV may amplify the risk of overfitting or become overly sensitive to outliers, thereby undermining the representation of model stability.

**Table 2:** Performance indicators for the random forest model.

Group	Precision [95% CI]	Sensitivity [95% CI]	F1 score [95% CI]	Specificity [95% CI]	AUC [95% CI]	PR-AUC
Group 1	0.790 [0.710, 0.923]	0.882 [0.750, 0.977]	0.833 [0.785, 0.911]	0.769 [0.585, 0.940]	0.905 [0.923, 0.977]	0.914
Group 2	0.771 [0.712, 0.923]	0.810 [0.628, 0.962]	0.790 [0.718, 0.893]	0.829 [0.703, 0.961]	0.901 [0.920, 0.977]	0.863
Group 3	1.000 [0.939, 1.000]	0.095 [0.000, 0.625]	0.174 [0.105, 0.769]	1.000 [0.995, 1.000]	0.992 [0.985, 1.000]	0.901

## Discussion

In this study, we categorized the perioperative blood glucose trajectories of patients undergoing TJA into three groups using GBTM. The blood glucose levels in the normal blood glucose group remained largely within the normal range. The group with slightly elevated blood glucose exhibited values that were essentially normal on the day prior to surgery, with a modest increase observed postoperatively. However, the overall fluctuations were relatively minor. In contrast, the hyperglycemic group maintained levels above the normal range both preoperatively and postoperatively, displaying considerable variability. Utilizing three groups of blood glucose trends as dependent variables, we constructed a RF model. The findings indicated that age, RBC levels one day post-surgery, hypertension, diclofenac, intraoperative blood transfusion volume, and the HEI as predictors of varying blood glucose trajectories in patients undergoing TJA during the perioperative period. However, the model’s recognition of Group 3 revealed low sensitivity, so these findings generate hypotheses regarding key predictors that warrant further validation.

Previous research has documented the occurrence of perioperative stress-induced hyperglycemia in patients undergoing total joint replacement (15). However, these studies included both diabetic and non-diabetic patients and did not clearly differentiate whether stress-induced hyperglycemia manifested before or after the surgical procedure. We excluded patients with diabetes mellitus. Whereas, 8.8% of the remaining patients exhibited hyperglycemia prior to surgery. The underlying cause may be either undiagnosed diabetes mellitus before hospitalization or stress-induced hyperglycemia following admission, warranting further investigation. Additionally, blood glucose volatility is recognized as a significant factor influencing perioperative outcomes. Patients presenting with preoperative hyperglycemia typically experience further increases in blood glucose levels post-surgery, which adversely impacts their rehabilitation outcomes. We should encourage these patients to undergo specialized diabetes diagnosis and treatment after discharge, in order to prevent or treat diabetes mellitus.

In this study all blood glucose measurements used fingertip rapid blood glucose testing. The point-of-care glucometry is known to exhibit greater variability compared with venous laboratory glucose (16). In the perioperative setting, peripheral perfusion may be compromised due to factors including hypothermia, vasoconstriction from anesthetic agents, and fluid shifts, potentially introducing blood glucose measurement bias, particularly during the early postoperative period (POD 0) (17, 18). This variability could influence the classification of patients at the boundaries between trajectory groups, where small differences in glucose values may shift group assignment. Nevertheless, capillary glucose monitoring remains the standard of care for perioperative glucose surveillance in most orthopedic wards, and our use of this method enhances the clinical applicability and generalizability of our findings. If multiple blood glucose tests were conducted within a short period of time, it is not practical to rely on the venous laboratory glucose. Future similar studies recommend the use of continuous glucose monitors.

Jia et al. (15) investigate the predictive value of the stress hyperglycemia ratio (SHR) for perioperative hidden blood loss (HBL) in patients with hip fractures. This study emphasizes a single blood glucose measurement, neglecting the dynamic nature of blood glucose fluctuations. Previous research has examined variations in perioperative blood glucose while a single trajectory inadequately represents the entire population, lacking a detailed analysis of patients across different subgroups (3, 7, 9). In contrast, GBTM facilitate the identification of latent clusters of patients exhibiting similar perioperative blood glucose trajectories, thereby enabling a more precise definition of specific patient groups that experience significant fluctuations in blood glucose.

The findings of this study suggest that age significantly influences the prediction of various blood glucose trajectories in TJA patients during the perioperative period. Previous research (19) has established a strong correlation between age and the incidence of postoperative stress-induced hyperglycemia. As age increases, the body’s metabolic regulation diminishes, leading to reduced blood glucose control, impaired responsiveness to stimuli, and an elevated risk of stress-induced hyperglycemia. Additionally, the volume of intraoperative blood transfusion and RBC levels on the first postoperative day serve as critical predictors. Jia et al. (15) demonstrated that the stress hyperglycemia ratio possesses predictive value for perioperative hidden blood loss in patients with hip fractures. A potential causal relationship may exist between surgical blood loss and fluctuations in blood glucose levels. However, the specific nature and underlying causes warrant further investigation.

Notably, this study identified the use of diclofenac during the perioperative period as a significant predictor of blood glucose trajectories, with the second-largest Lasso coefficient (−0.3098) among the six retained variables and ranking 4th in RF variable importance. This finding has not been previously reported and merits careful pharmacological consideration. Diclofenac is a non-selective cyclooxygenase (COX) inhibitor that suppresses the synthesis of prostaglandins and thromboxanes from arachidonic acid. In the perioperative context, surgical tissue injury triggers a cascade of inflammatory mediators. These neuroendocrine hormones are primary drivers of stress-induced hyperglycemia, enhancing hepatic gluconeogenesis, promoting glycogenolysis, and increasing peripheral insulin resistance (20). By attenuating the COX-mediated inflammatory cascade, diclofenac may blunt the subsequent neuroendocrine stress response, thereby moderating the cortisol and catecholamine surges that contribute to perioperative blood glucose elevation. This represents a biologically coherent mechanism by which NSAID administration could influence glucose trajectories. However, the observed association between diclofenac use and more favorable glucose trajectories must be interpreted with caution, as it may partly reflect confounding by indication. Because the above-mentioned mechanism of action is also influenced by the severity of the disease. Disentangling these two mechanisms would require more granular data on surgical complexity, pain scores, and dosage-timing profiles of diclofenac administration, which were not systematically collected in the present study. Future prospective studies employing propensity score matching or instrumental variable approaches are warranted to isolate the independent effect of diclofenac on perioperative glucose dynamics.

Combined hypertension is a significant predictor of varying blood glucose trajectories in TJA patients during the perioperative period. Several factors contribute to this analysis. First, hypertension is often associated with increased insulin resistance, which can disrupt normal blood sugar metabolism. Second, heightened sympathetic nervous system activity may impair pancreatic function and diminish insulin secretion. Additionally, negative emotions, such as anxiety and depression, can elevate the risk of stress-induced hyperglycemia. These adverse emotional states can significantly impair the body’s endocrine function, enhance gluconeogenesis and cortisol secretion, promote excessive norepinephrine and epinephrine release, increase insulin resistance, and ultimately lead to elevated blood glucose levels.

This study revealed three heterogeneous blood glucose trajectories among TJA patients during the perioperative period, indicating that a one-size-fits-all glucose management strategy is insufficient and supporting the need for trajectory-based stratified monitoring. Second, age, postoperative RBC, hypertension, diclofenac, intraoperative blood transfusion volume, and HEI were identified as key predictors, providing monitorable and modifiable references for preoperative risk assessment that warrant clinical attention. Third, although the model has limited ability to identify hyperglycaemic trajectories, the trajectory classification itself and the identified predictors hold value for screening and early warning, laying the groundwork for future prospective validation with larger samples and balanced class distributions.

### Strengths and limitations

The primary strength of the current study lies in the application of GBTM to TJA patients, enabling the identification of specific patterns in blood glucose trajectories over time, as well as the characteristics associated with these distinct trajectories. However, the study is limited by its small sample size, which may influence the estimated shapes and classifications of the trajectory groups. Future research with larger sample sizes is essential to replicate these findings. Additionally, the current study did not track the rehabilitation outcomes of patients categorized by their blood glucose trajectories. Future prospective studies should incorporate postoperative outcome assessment on the basis of reasonable sample size estimation and rigorous design, in order to verify the clinical significance of the classification of blood glucose trajectories and determine whether the intervention based on trajectory grouping can improve surgical outcomes. Furthermore, we routinely implemented hypoglycemic interventions and did not control for the effects of medications, such as analgesics, in patients with preoperative hyperglycemia, which may have influenced the trajectories observed. Nonetheless, this approach may render our results more reflective of real-world conditions. Finally, the relationships between patient predictors identified by the RF model require external validation in future studies. Finally, the RF model exhibited markedly low sensitivity for the hyperglycemia trajectory group (Group 3). Given that this subgroup represents the population at greatest clinical risk for adverse perioperative outcomes, the high false-negative rate constitutes a significant barrier to clinical translation. The model’s apparent high AUC for Group 3 was substantially inflated by class imbalance and should not be interpreted as evidence of adequate discriminative performance. It may have constrained the RF algorithm’s ability to learn distinguishing features for this minority class. Future studies should consider targeted recruitment of patients with elevated blood glucose to address this imbalance. Consequently, the present model should be regarded as exploratory and its clinical application value requires further research.

## Conclusion

Based on the findings of this study, we identified three subgroups of perioperative blood glucose trajectories in TJA patients and analyzed their predictors using a RF model. A preliminarily understanding of blood glucose trajectories in TJA patients may facilitate a personalized approach to perioperative blood glucose management for this population and beyond. However, at the current classification threshold, the model has limited ability to identify patients with hyperglycaemic trajectories. Future prospective studies with balanced class representation are needed before clinical application can be considered.

## Data Availability

The original contributions presented in the study are included in the article/Supplementary material, further inquiries can be directed to the corresponding author.
