# Supplementary material for: The blood glucose trajectories among non-diabetic patients with total joint arthroplasty: clinical characteristics and predictors
Source: Front Med (Lausanne). 2026 Jun 25;13:1820392. doi: 10.3389/fmed.2026.1820392 (PMC13346176; doi:10.3389/fmed.2026.1820392)

Supplementary Table 1. Comparison of Information Criteria across Competing Models

Group	AIC	BIC	CAIC	SSBIC	HQIC
1	14938.65	14969.59	14974.59	14953.70	14949.67
2	14044.70	14112.77	14123.77	14077.82	14068.96
3	13831.66	13936.86	13953.86	13882.85	13869.15
4	13796.70	13939.04	13962.04	13865.95	13847.42
5	13799.68	13979.15	14008.15	13887.01	13863.60

Note. AIC = Akaike Information Criterion; BIC = Bayesian Information Criterion; CAIC = Consistent Akaike Information Criterion; SSBIC = Sample-Size Adjusted BIC; HQIC = Hannan-Quinn Information Criterion. Smaller values indicate better model fit.

Supplementary Table 2. The evaluated results of model using LOOCV

Metric	Original Model	LOOCV Model
Overall Accuracy	0.7833	0.5333
Kappa Coefficient	0.6004	0.135
Class: Group1 - Sensitivity	0.8824	0.6471
Class: Group1 - Specificity	0.7686	0.5041
Class: Group1 - Precision	0.7895	0.562
Class: Group1 - F1 Score	0.8333	0.6016
Class: Group2 - Sensitivity	0.81	0.51
Class: Group2 - Specificity	0.8286	0.6357
Class: Group2 - Precision	0.7714	0.5

Metric	Original Model	LOOCV Model
Class: Group2 - F1 Score	0.7902	0.505
Class: Group3 - Sensitivity	0.0952	0
Class: Group3 - Specificity	1	0.9954
Class: Group3 - Precision	1	0
Class: Group3 - F1 Score	0.1739	NA

Supplementary Figure 1. Trajectory plots of 4-group solutions identified by GBTM. Percentages indicate the proportion of patients assigned to each trajectory group.

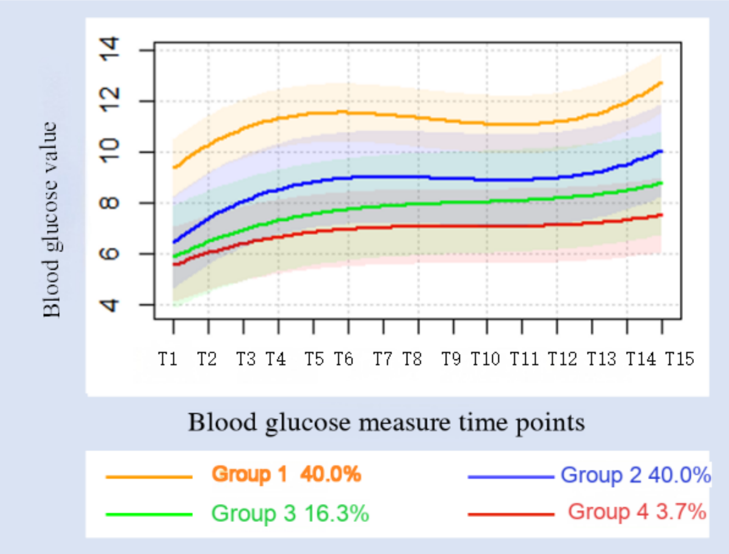

Supplement: Supplementary file 1 [file Table_1.pdf]
